# Bu Yang Huan Wu Decoction controls synovitis via HIF-1/VEGF signaling for osteoarthritis therapy

**DOI:** 10.1097/MD.0000000000045375

**Published:** 2025-10-31

**Authors:** Xue Li, Ying Lv, Xingyong Yang, Siyu Wang, Qi Liang

**Affiliations:** a Department of Clinical Laboratory, Affiliated Hospital of North Sichuan Medical College, Nanchong, Sichuan Province, China; b Department of Clinical Laboratory, Chengdu Integrated TCM & Western Medicine Hospital, Chengdu, Sichuan Province, China; c Intensive Care Unit, Affiliated Hospital of North Sichuan Medical College, Nanchong, Sichuan Province, China.

**Keywords:** arthritis, computer-assisted, herbal medicine, hypoxia, therapy

## Abstract

Articular cartilage deterioration is a hallmark of osteoarthritis (OA). Articular cartilage is structurally destroyed as a result of mechanical, metabolic, and inflammatory factors that are part of the etiology of OA. Although the precise molecular processes in OA are still unclear, the hypoxic microenvironment is crucial. For the clinical therapy of orthopedic illnesses, particularly OA, the Bu Yang Huan Wu Decoction (BYHWD) is frequently utilized, however, the precise pharmacological mechanism behind its action is unclear. With the use of transcriptome and network pharmacology, we want to understand the molecular mechanisms behind OA and the pharmacological mechanism of BYHWD for OA treatment. The traditional Chinese medicine database and incoming blood component data were used to assess the therapeutic components and targets of action of BYHWD, while the transcriptome data was used to analyze the critical targets and possible immunological and inflammatory mechanisms of OA occurrence. Using gene ontology and Kyoto encyclopedia of genes and genomes, additional research was conducted based on the network pharmacological analysis to determine the biological mechanism of BYHWD for treating OA. Eight active components of BYHWD were found by drug screening, astragaloside, kaempferol, formononetin, and paeoniflorin may be significant players. The HIF-1/VEGF signaling way, EGFR tyrosine kinase inhibitor resistance, and Rap1 signaling pathway are the primary biological processes involved in BYHWD treatment OA. BYHWD reduces synovitis by taking part in HIF-1/VEGF signaling, which controls immune and inflammatory factors through important components like formononetin, kaempferol, paeoniflorin, astragaloside, etc. This offers a fresh perspective on treating OA and applying traditional Chinese medicine.

## 1. Introduction

Degeneration and loss of articular cartilage, along with bone regeneration in the articular rim and subchondral bone, are the hallmarks of osteoarthritis (OA), a chronic joint disease that starts in the articular cartilage.^[1]^ Clinical symptoms that are frequently seen include joint discomfort, swelling, decreased mobility, etc; in later stages, these symptoms may result in joint deformity or even disability.^[2]^ According to epidemiological research, 17% of those over 40 have OA overall, and as society ages, this prevalence will progressively rise.^[2,3]^ The pathophysiology of OA entails the involvement of mechanical, metabolic, and inflammatory processes that result in joint structural degradation and functional alterations. It has been proposed recently that instead of the typical passive degenerative or wear-and-tear illnesses, OA is caused by an imbalance between joint tissue destruction and repair, leading to active dynamic alterations. Numerous factors, including inflammation, biomechanics, excessive chondrocyte death, autoimmunity, and inheritance of susceptibility genes, are linked to this imbalance.^[4]^ Chinese and Western medicine are currently primarily used in tandem to treat OA. Recent advances in medical technology have made it possible to diagnose and treat certain orthopedic diseases more accurately and completely. However, there are still issues with modern medical procedures, a lack of specialized therapies, and a lack of a thorough understanding of the pathogenesis and pathological process of these conditions.^[5]^ “Bone paralysis,” “pain paralysis,” “calendar disease,” and other terms are used to describe OA. The principal causes of its etiology are the loss of tendons and veins, liver and kidney deficiencies, and age-related blood and qi shortages. The primary causes of the illness are blood and qi shortages brought on by aging, liver and kidney deficiencies, tendons and vein deficiencies, and inflammation of the veins and channels from the outside wind, cold, and moisture. In recent years, the classic Chinese medicine formula of Bu Yang Huan Wu Decoction (BYHWD) has begun to be applied in orthopedic clinics, which has better efficacy and lower adverse effects on certain orthopedic diseases, and therefore has gradually attracted the attention of scholars and clinicians.^[6–8]^

There is mounting evidence to support the theory that hypoxia and hypoxia-inducible factor 1α (HIF-1α) are important players in the pathological angiogenesis process. A crucial downstream effector molecule of HIF-1α, vascular endothelial growth factor-α (VEGF-α), can trigger endothelial cell activation, proliferation, and migration, leading to neovascularization.^[9]^ Thus, inhibiting the activation of the HIF-1α/VEGF-α signaling pathway could potentially improve the pathogenic neovascularization associated with OA.^[10]^ One of the pathological characteristics of OA is synovitis, and the joint cavity’s hypoxic microenvironment is the condition it is in. The hypoxia in the joint cavity of OA is impacted and made worse by the ongoing expansion of synovium in the joint cavity as well as the high energy consumption of synovial inflammation. When cells are in a hypoxic milieu, such as in the synovial tissues of OA patients, hypoxia enhances the expression of HIF-1α, which is also favorably correlated with the number of blood vessels and inflammatory cell infiltration. These comprise VEGF, interleukin (IL)-1, TNFα, and so on.^[11]^ A major contributing element to synovitis in OA is the hypoxic microenvironment, which also triggers the invasion of inflammatory cells and the synthesis of inflammatory substances that boost the local immune response. The migration of synovial membranes in OA is caused by the direct regulation of matrix metalloproteinases (MMPs) by HIF-1α, which also influences their secretion. By inhibiting the hypoxia-induced synovial invasive pathological condition of OA, HIF-1α blockers can reduce synovial inflammation.^[12,13]^ Consequently, one possible method for treating OA is to modify the HIF-1/VEGF signaling system.

Advances in the life sciences have been made possible by a novel approach to illness research in recent years, based on transcriptome sequencing-based analysis of bioinformatic processes.^[14,15]^ Herbal network pharmacology is also still a simple approach to research how medications and illnesses are related. Ocular pathology data, particularly those related to OA, are becoming more abundant. These data may be acquired through the Gene Expression Omnibus (GEO) database, allowing for a thorough examination of the mechanisms behind the development of OA.^[16]^ Our goal in this work is to gather transcriptome information on OA in order to examine any possible molecular causes. Furthermore, using data from Chinese medicine databases and blood component analyses, we will examine the active ingredients and molecular mechanisms of BYHWD for the treatment of OA in more detail. Our research aims to further TCM development, clarify the illness molecular mechanism of OA, and investigate the role of BYHWD in OA treatment.

## 2. Materials and methods

### 2.1. Drug data collecting by BYHWD

An internet resource called the traditional Chinese medicine systems pharmacology (TCMSP, https://tcmspw.com/tcmsp.php) compiles the chemical makeup of popular herbal remedies. This database systematically integrates the chemical composition of traditional Chinese medicine (TCM), ADME (absorption, distribution, metabolism, excretion) properties, target of action, and related disease information, providing a standardized, quantifiable, and reliable platform for efficient screening of potential active ingredients and their molecular targets in TCM formulas. It is currently the most commonly used database for TCM prescriptions and has the most abundant data information, so it is often chosen. The herbal remedies Huangqi (Hedysarum Multijugum Maxim.), Danggui (Angelicae Sinensis Radix), Chishao (Radix Paeoniae Rubra), Dilong, Chuanxiong (Chuanxiong Rhizoma), Honghua (Carthami Flos), Taoren (Persicae Semen) are registered in the database under the BYHWD designation. Based on oral bioavailability ≥ 30% and drug-likeness ≥ 0.18, compounds were gathered for each medication.^[17,18]^ A drug-chemical composition network map of BYHWD was created using Cytoscape software. Database of Constituents Absorbed into Blood and Metabolites of Traditional Chinese Medicine (DCABM-TCM; dcabm-tcm [ncpsb.org.cn]), a DCABM-TCM, is the first database systematically collecting blood constituents of TCM prescriptions and herbs.^[19]^ This database was used to gather verified incoming blood components for BYHWD, and Cytoscape was then used once more to create a network of arriving blood components for BYHWD. Using Venny diagrams, the compositional intersection of 2 databases – TCMSP and DCABM-TCM – was created in order to identify the main active chemicals in BYHWD. Pubchem is a data library of substances where users may find out fundamental details about common compounds (https://pubchem.ncbi.nlm.nih.gov/). Gather the SMILES numbers and chemical structure formulas for the main BYHWD active components. The database at Swiss Target Prediction (http://www.swisstargetprediction.ch/?) combines duplicates to determine final targets and employs SMILES to forecast targets of action for important chemicals.^[20]^

### 2.2. Analysis of data on osteoarthritis

We gathered transcriptome data GSE55457 for an OA analysis and GPL96 as a sequencing platform for sample data from the GEO database, an online resource that offers transcriptome data for a variety of disorders.^[21]^ In this work, Sangerbox 3.0’s R language tool set was used to examine the data. Prior to being submitted to an online tool, the raw data were first retrieved, their redundant information deleted, and their gene names labeled. The sample data were divided into statistical groups by the Rtsen program, which then created a UMAP downscaling map of the data. The “limma” software package performed overall differential gene screening on the processed data, with a screening criterion of *P* < .05 and a difference multiple log2 (FoldChange) greater than or equal to 0.5. The results were presented in the form of volcano or heatmap.^[22]^

An extensive, searchable, and free database of predicted and annotated human genes is available at GeneCards (https://www.genecards.org). Gene-centered data, comprising genomic, transcriptomic, proteomic, genetic, clinical, and functional information, are automatically integrated by the knowledge base from about 150 web resources. To find the target proteins of OA, we conducted a database search with the phrase “arthritis.”^[23–25]^

### 2.3. BYHWD pivotal gene screening for the treatment of OA

Based on Genecards, we were able to identify targets for OA data, major differential genes in the process of OA occurrence, and treatment targets for key molecules of BYHWD through preliminary study. In order to discover the key genes for BYHWD treatment of OA, we intersected the above data, including BYHWD drug targets, arthritis Genecards disease targets, and differential gene targets in transcriptome data GSE57554. This intersection gene set is the potential therapeutic target. Protein–protein interactions, also referred to as PPIs for short, are the building blocks of protein interaction networks, which are made up of interactions between proteins that facilitate the activation of key regulatory genes. Proteins with high connectedness may be essential for metabolism or signaling, while highly aggregated proteins may share identical or comparable functions. We performed a PPI network analysis on the set of intersecting genes, which was limited to “*Homo sapiens*,” with a confidence value > 0.4, using the string database (http://string-db.org/). A plug-in for the Cytoscape program called the CytoHubba algorithm was also used to exclude genes that lacked apparent connections. Additionally, irrelevant genes were eliminated using the CytoHubba algorithm, a plug-in for the Cytoscape program. Sort by degree value, with the top 4 genes constructed using red diamonds, and the rest constructed using a circular network graph based on sorting and color depth. The co-expression of the hub genes is displayed as a heatmap of co-expression scores derived from RNA expression patterns and protein co-regulation. We identified the pivotal genes for OA treatment by pivotal gene analysis, and we statistically analyzed the top 10 data genes to find their expression in OA. Using the gene names in GSE57554, the expression of several tissue samples was retrieved, and bar graphs were used for statistical analysis. The data was analyzed using GraphPad Prism 10 software, and the results were expressed as *x* ± *s*. The comparison between the 2 groups was conducted using *t*-test, with *P < .05, **P < .01, and ***P < .001 indicating statistically significant differences.

### 2.4. Analysis of bioprocesses based on possible treatment targets

Open Ouyi BioCloud, import the crucial gene of BYHWD OA, choose gene ontology (GO), Kyoto encyclopedia of genes and genomes (KEGG), and Wikipathways enrichment analysis, and restrict the species to “*H sapiens*.” In the parameters, type the therapeutic target’s gene symbol and hit submit. Several graphs display the bioprocess’s end outcomes. The data of drugs, key components, intersecting genes and hub genes of BYHWD for OA were imported into Cytoscope software. After calculation, the effect network diagram of “BYHWD-drug-active compound-cross-cutting gene-OA” was constructed.^[26]^

### 2.5. Immunoassay for OA

The percentage of 6 different immune cell types (DC, NK, B, macrophage, neutrophil, and monocyte cells) as well as the fraction of 18 T cells can be estimated by immune cell AI. Immune Cell AI (ICA) integrates multi-omics data (e.g., scRNA-seq, epigenetics, spatial transcriptomics) with a context-aware deep learning framework, enabling higher-resolution deconvolution of immune cell subsets (e.g., exhausted T cells, M2 macrophages) in complex tissues. Unlike bulk-tool-centric methods (e.g., CIBERSORTx, xCell), ICA dynamically adapts to tissue-specific microenvironments and mitigates batch effects, significantly improving accuracy in disease-specific contexts. Additionally, if group data is available, it can use the conventional ANOVA test to evaluate the variations in the proportions of individual immune cells across groups and forecast the patient’s reaction to immune checkpoint inhibitor medication.^[27]^ We computed the immune cell types of patients with varying immunization patterns in the transcriptome dataset using the Immune Cell AI algorithm in the BioSignal Beanstalk program. To illustrate the immune CC of the patients, we utilized stacked graphs. The precise visualization of immune cell infiltration in various sample subgroups was achieved through the use of immune cell radar plots, while correlation dot-bar plots were employed to identify correlations in immune cell infiltration.

## 3. Results

### 3.1. Results of BYHWD medication data

**Figure 1.**
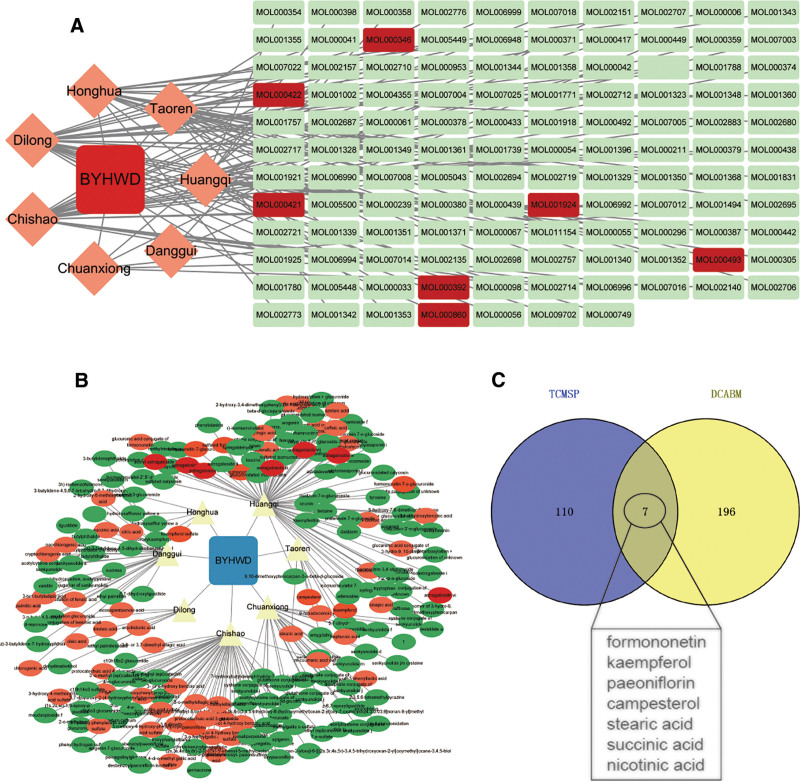
(A) BYHWD’s drug components are mainly from TCM data, (B) BYHWD’s drug entry components are mainly from entry TCM data, and (C) BYHWD’s drug data intersect with entry components. BYHWD = Bu Yang Huan Wu Decoction, DCABM = database of constituents absorbed into blood and metabolites, TCM = traditional Chinese medicine, TCMSP = traditional Chinese medicine systems pharmacology.

**Figure 2.**
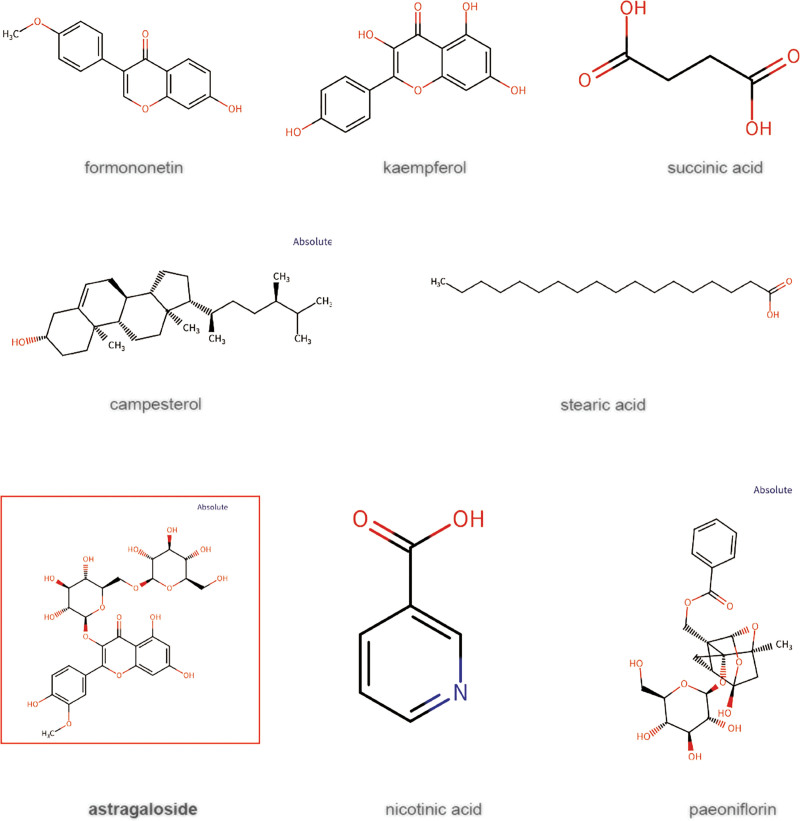
Chemical structures of key drug components of BYHWD. BYHWD = Bu Yang Huan Wu Decoction.

We found 134 medicinal chemical elements of BYHWD in the TCMSP database based on the predetermined screening criteria; however, Dilong was not included in the database, therefore we used ITCM data to complement it. We eventually acquired 117 online TCMSP database of BYHWD medications after deleting duplicate screening by the same components of different drugs (Table S1, Supplemental Digital Content, https://links.lww.com/MD/Q557, Fig. 1A). Blood entry components of common herbs are provided by DCABM-TCM. Through database search, we were able to locate 250 blood entry compounds for BYHWD. Similarly, eliminating duplicates produced 203 blood entry components in the end (Table S2, Supplemental Digital Content, https://links.lww.com/MD/Q557, Fig. 1B). 8 final essential chemicals were obtained by the intersection of TCM’s blood entry components and compounds: formononetin, kaempferol, paeoniflorin, campesterol, stearic acid, succinic acid, and nicotinic acid and astragaloside. Furthermore, we discovered that astragaloside, the blood entry component of Huangqi, which is the medication that primarily treats BYHWD, was highly expressed. In the end, we determined that these 8 chemicals were the primary active ingredients in BYHWD (Fig. 2). A total of 424 effective targets of action for BYHWD were ultimately determined when the targets of pharmacological action for the primary active components were predicted (Table S3, Supplemental Digital Content, https://links.lww.com/MD/Q557). Kaempferol and paeoniflorin inhibit PTGS2, block prostaglandin E2 production, and alleviate inflammatory pain; Huangqi IV glycoside regulates the PTPRC pathway, inhibits abnormal activation of T/B cells, and improves immune homeostasis. Succinic acid and niacin regulate IGF1R-PI3K/AKT, improve chondrocyte metabolism, and inhibit apoptosis. In summary, this study integrated the TCMSP and ITCM databases to screen out 8 core active ingredients (kaempferol, paeoniflorin, niacin, and astragaloside IV) and their targets of Buyang Huanwu Tang. These ingredients and targets intervene in the core pathological process of OA through a synergistic mechanism.

### 3.2. Differential gene analysis examining the course of arthritis disease

**Figure 3.**
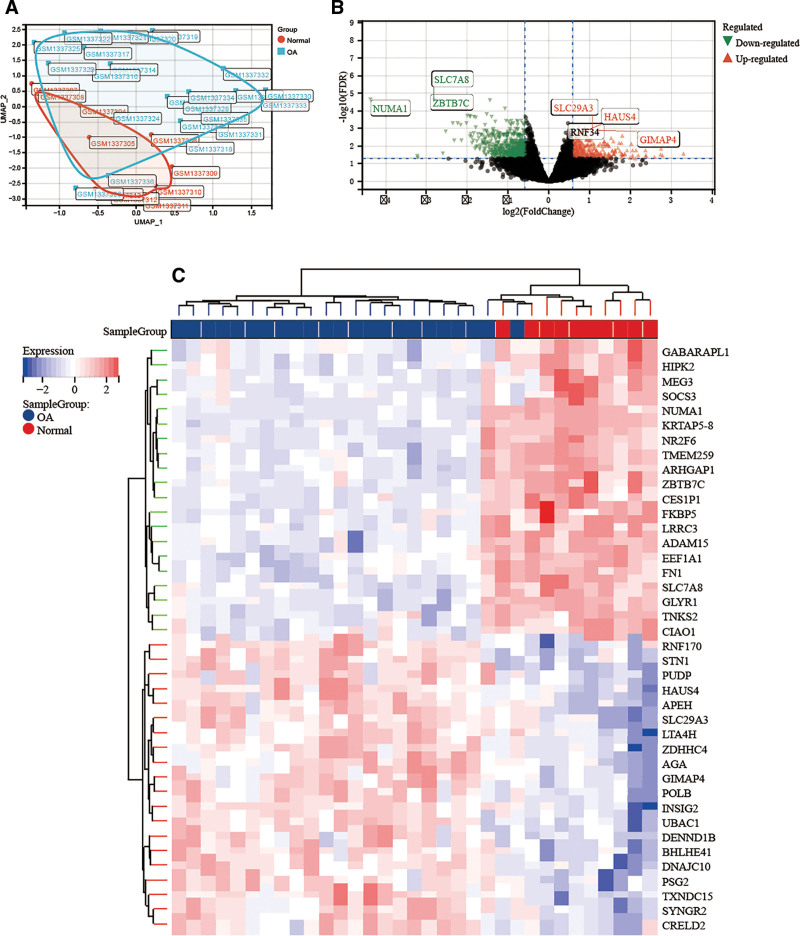
(A) Arthritis transcriptome data de-batched, (B) differential volcano plots of arthritis transcriptome data, (C) differential heat maps of arthritis transcriptome data.

Following the processing of the GSE55457 data, we were able to identify 23 OA tissues and 10 normal tissues for examination. The normal and OA tissues are initially shown to be distributed in distinct quadrants and to partially intersect on the UMAP plots of the downscaled analysis. This indicates that the sample tissues are distinct and have intricate connections, and as such, the sample tissues can be examined (Fig. 3A). More than 12,000 genes are expressed in the tissue according to a differential study of GSE55457, and limma difference analysis provides a broad picture of gene expression. Genes with no discernible alterations are shown by black color, down-regulated genes are shown by green color, and up-regulated genes are shown by red color (Tables S4 and S5, Supplemental Digital Content, https://links.lww.com/MD/Q557, Fig. 3B). It revealed that SLC7A, NUMA1, and ZBTB7C belonged to considerably down-regulated genes, and SLC29A3, HAUS4, and GIMAP4 belonged to significantly up-regulated genes among them. Lastly, a differential genes heat map (Fig. 3C) further illustrates genes with notable expression differences by color-coding up-regulated and down-regulated genes.

### 3.3. Crucial therapeutic targets for BYHWD in the management of arthritis

**Figure 4.**
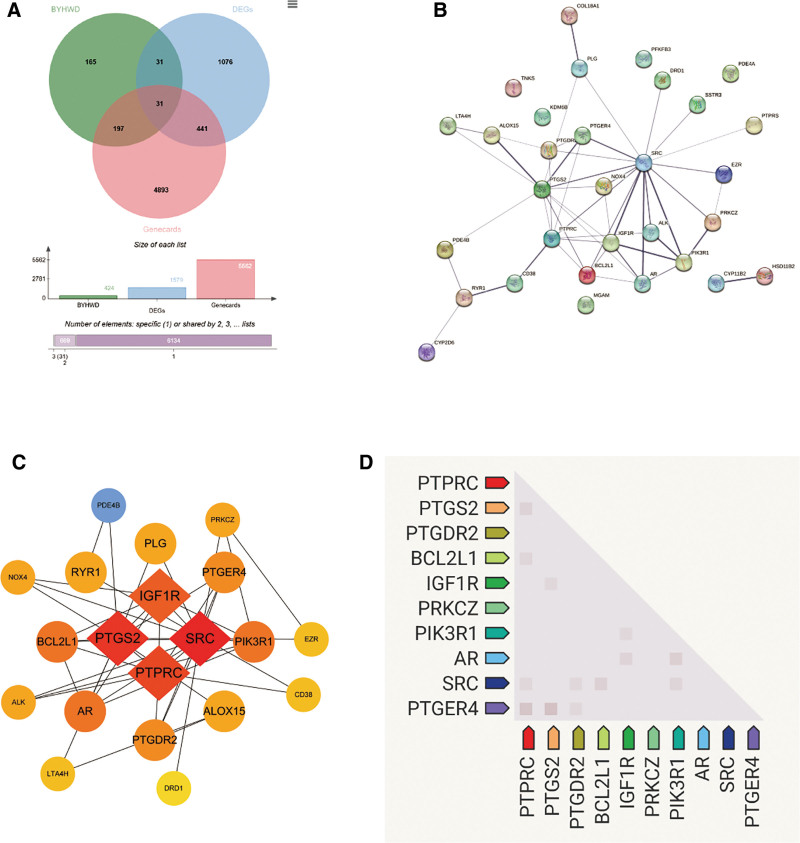
(A) Target intersection of BYHWD with transcriptome differential genes, Genecards, (B) PPI network map of intersected targets, (C) Protein interactions map with unassociated genes removed, (D) Co-expression pattern of the top 10 pivotal genes. BYHWD = Bu Yang Huan Wu Decoction, PPI = protein–protein interaction.

424 active ingredient action targets for BYHWD, 1579 differentially expressed genes for OA tissue samples, and 5562 OA disease genes from Genecards were obtained through the analysis. Thirty-one genes intersected at the target intersection of the 3 data (Table S6, Supplemental Digital Content, https://links.lww.com/MD/Q557, Fig. 4A). The PPI network showed the therapeutic targets’ interaction network, of which TNKS, PDE4A, PFKFB3, and CYP11B2 were eliminated without showing any correlation with other targets. Using the degree ordering and Cytoscape’s computations, a network diagram of the remaining targets was once more created; the color hues indicate how important each target is to the network (Table S7, Supplemental Digital Content, https://links.lww.com/MD/Q557, Fig. 4B, C). SRC, PTGS2, PTPRC, and IGF1R were BYHWD’s primary OA action targets. As an inflammatory signaling hub, SRC is activated by cytokines such as IL-6/TNF-α, phosphorylating the PI3K/AKT and MAPK pathways, promoting abnormal proliferation, invasion, and secretion of matrix metalloproteinases (MMPs) in synovial cells, directly damaging articular cartilage. Highly expressed under inflammatory stimulation, PTGS2 catalyzes the synthesis of prostaglandin E 2 (PGE2) from arachidonic acid, mediates synovial vasodilation, edema, and pain sensitization, and activates osteoclasts leading to bone erosion. PTPRC, as a co regulatory molecule of T/B cell receptors, regulates lymphocyte activation threshold by dephosphorylating Lyn/Fyn kinase; its abnormal expression can break immune tolerance, promote the infiltration of self reactive lymphocytes into joints, and drive autoimmune arthritis. In OA, chondrocytes stress upregulate IGF1R in an attempt to repair, but sustained activation induces excessive PI3K mTOR signaling, which accelerates chondrocyte metabolic disorders, aging, and apoptosis, and promotes synovial vascular opacities to invade cartilage. Ultimately, the co-expression heatmap of the top 10 crucial genes was created using the gene co-expression pattern map as a basis. The findings demonstrated a substantial relationship between PTPRC and SRC, PTGER4, BCL2L1, and PTGS2, a significant relationship between PTGS2 and IGF1R, and a significant relationship between BCL2L1 and PIK3R1 and AR.

**Figure 5.**
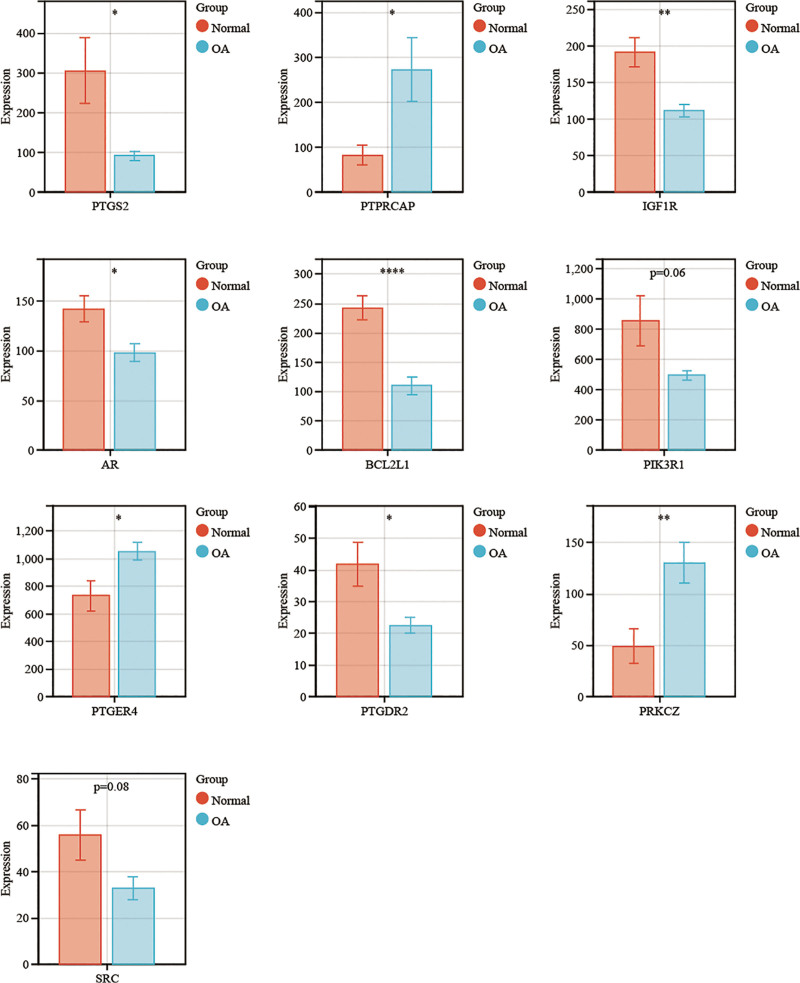
Statistical analysis of the top 10 pivotal genes (*P < .05, **P < .01, ***P < .001). OA = osteoarthritis.

Following the screening of BYHWD’s important targets for the therapy of arthritis, we used statistics to examine the pivotal genes’ expression. The histogram demonstrated that the majority of the highly expressed genes in OA were PTGS2, IGF1R, AR, BCL2L1, PTGDR2, PIK3R1, and SRC. Of these, PTGS2, IGF1R, AR, BCL2L1, and PTGDR2 were statistically significant (2 sample *t*-test, *P < .05, **P < .01, ***P < .001). In contrast, 3 targets showed decreased expression in OA: PTPRCAP, PTGER4, and PRKCZ. These 3 targets were statistically significant (two sample *t*-test, *P < .05, **P < .01, ***P < .001; Fig. 5). The pharmacological active ingredients of Buyang Huanwu Tang reverse the core pathological phenotype of OA by synergistically regulating the 3 major hub target networks of SRC-PTGS2 (inflammation), PTPRC (immunity), and IGF1R (degeneration). Its multi-target mode of action provides a scientific basis for the treatment of complex arthritis with TCM formulas.

### 3.4. The role of biological processes in BYHWD arthritis treatment

**Figure 6.**
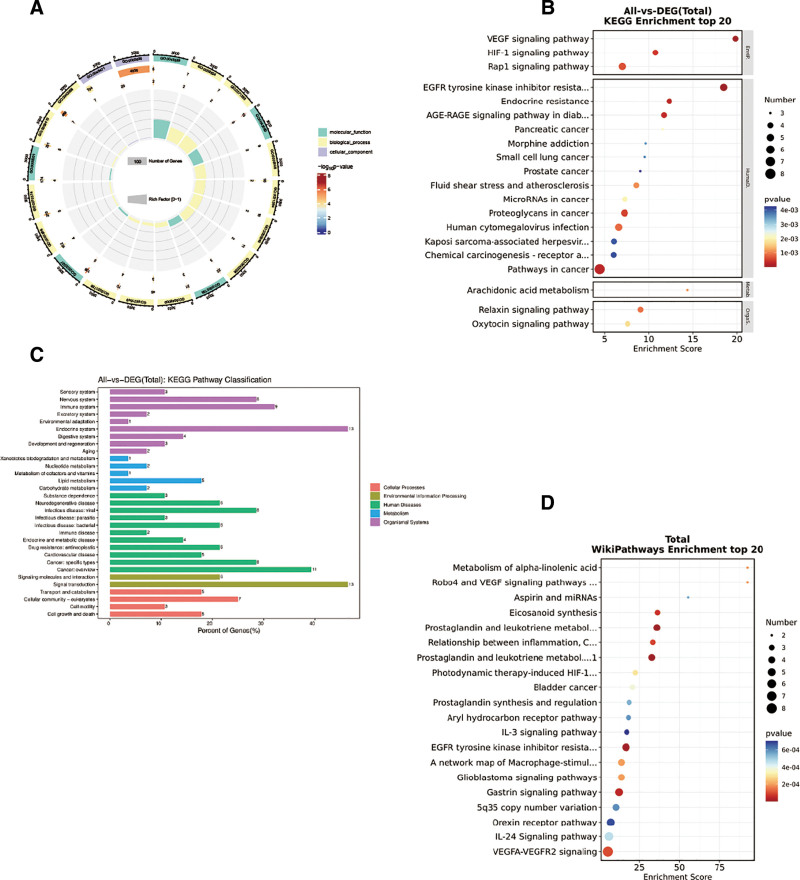
(A) Circle plot of GO enrichment analysis of the intersection genes, (B) bubble plot of KEGG enrichment analysis of the intersection genes, (C) categorical bar graph of KEGG enrichment analysis of the intersection genes, (D) bubble plot of WikiPathways enrichment analysis of the intersection genes. GO = gene ontology, KEGG = Kyoto encyclopedia of genes and genomes.

We were able to look into the biological mechanism of BYHWD treatment for OA further through the enrichment analysis of possible therapeutic targets. The GO analysis results showed that a total of 944 data points were obtained for the therapeutic targets, which were a summary of all biological processes (BP), cellular components (CC), and molecular function (MF) during the GO enrichment process. Among them, there are 652 BP data, 133 CC1 data, and 159 MF data (Table S8, Supplemental Digital Content, https://links.lww.com/MD/Q557, Fig. 6A). The results indicate that BYHWD treatment of the OA process is related to the insulin-like growth factor receptor signaling pathway, the cellular response to progesterone stimulation, the cAMP catabolic process, the cellular response to xenobiotic stimulation, the cellular response to apoptotic process, and the response to xenobiotic stimulation. Of these, the first ten results in BP, CC, and MF are displayed in chordal diagrams. The cytoplasmic extranuclear area, fossa, extracellular vesicles, choriocapillaris, ciliated membranes, glutamatergic synapses, nonmotile cilia, foci of adhesion, and the lumen of platelet alpha granules are the primary cellular locations of these targets. Various biochemical pathways, including heme, insulin, heparin, signaling receptor, phosphatidylinositol 3-kinase, 3′,5′-cyclo-AMP phosphodiesterase activity, ATPase, and asparagine sulfate proteoglycan binding, may be involved in the therapeutic process.

According to KEGG’s findings, BYHWD treatment of the OA process involves the VEGF signaling pathway, HIF-1 signaling pathway, EGFR tyrosine kinase inhibitor resistance, endocrine resistance, AGE-RAGE signaling pathway in diabetic complications, Rap1 signaling pathway, human cytomegalovirus infection, relaxin signaling pathway, fluid shear stress and atherosclerosis, arachidonic acid metabolism, Oxytocin signaling pathway, Morphine addiction (Table S9, Supplemental Digital Content, https://links.lww.com/MD/Q557, Fig. 6B, C), which is linked to the endocrine system, immunological system, signaling, cell development and death, and other processes.

**Figure 7.**
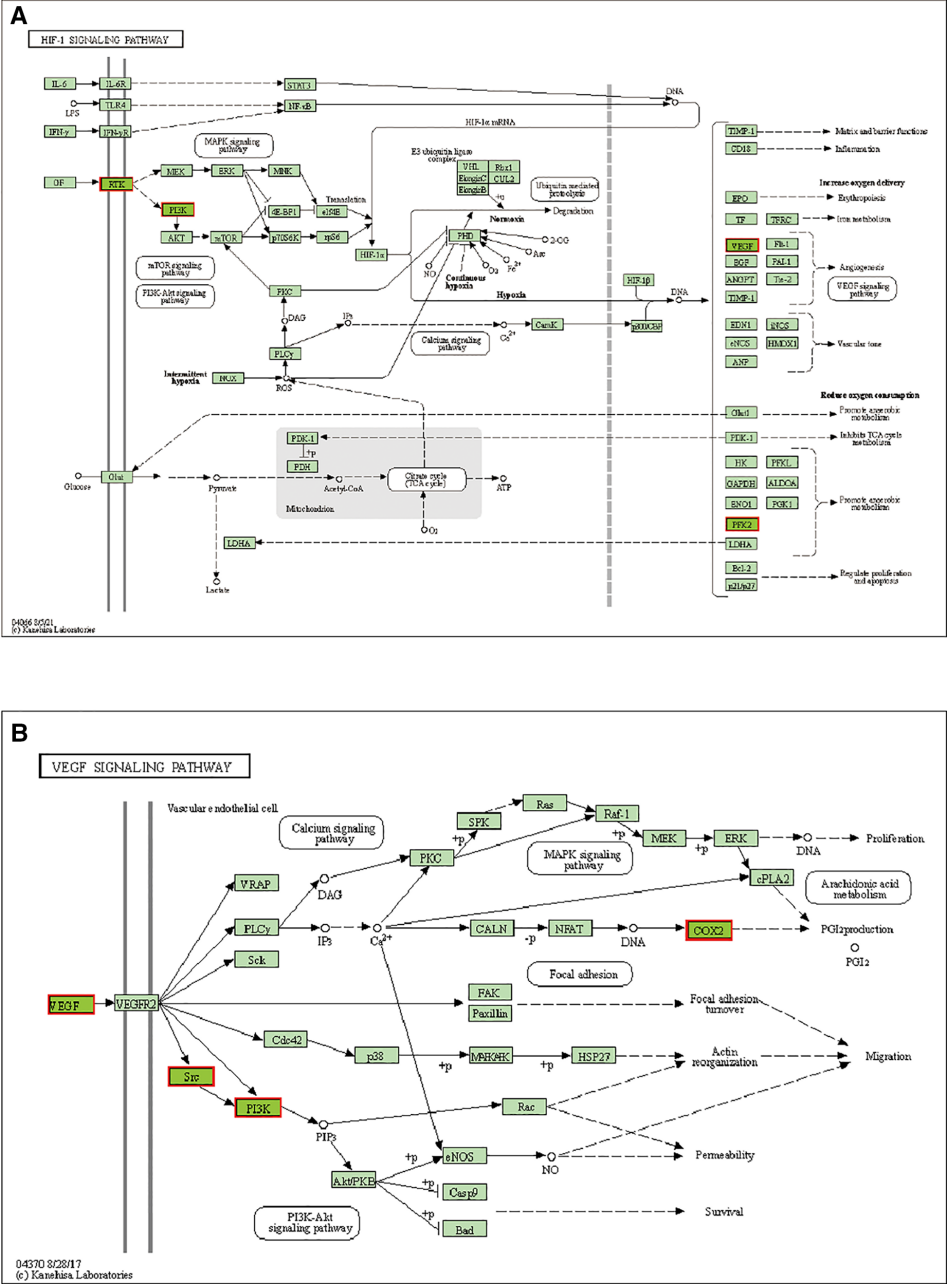
(A) HIF-1 signaling pathway from KEGG, (B) VEGF signaling pathway from KEGG. HIF-1 = hypoxia-inducible factor 1α, KEGG = Kyoto encyclopedia of genes and genomes, VEGF = vascular endothelial growth factor.

**Figure 8.**
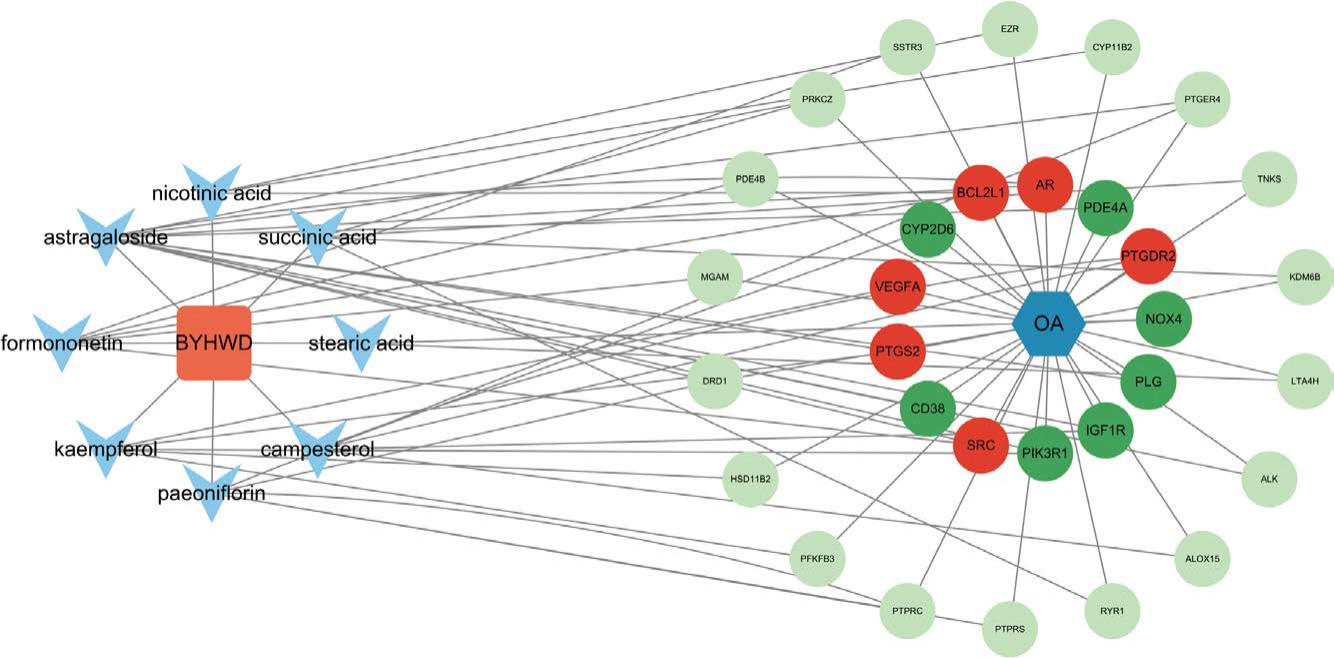
Network diagram of BYHWD for arthritis treatment. BYHWD = Bu Yang Huan Wu Decoction.

Lastly, the Wikipathway database contained a wealth of analogous signaling pathways, including those involved in the metabolism of prostaglandin and leukotrienes during senescence, prostaglandin and leukotriene metabolism in senescence, EGFR tyrosine kinase inhibitor resistance, eicosanoid synthesis, relationship between inflammation, COX-2 and EGFR, VEGFA-VEGFR2 signaling, metabolism of alpha-linolenic acid, Photodynamic therapy-induced HIF-1 survival signaling, IL-24 signaling pathway, IL-3 signaling pathway (Fig. 6D). Lastly, we examined the role that the HIF-1 signaling pathway and VEGF played in the OA treatment process (Fig. 7). Furthermore, we ultimately created a “BYHWD-drug-active compound-intersecting gene-OA” effect network diagram using computation (Fig. 8). In summary, the enrichment analysis of BYHWD in the treatment of OA reveals its synergistic effect through multidimensional pathways: the VEGF/HIF-1 signaling axis regulates synovial angiogenesis and hypoxic injury, and together with arachidonic acid metabolism, amplifies the inflammatory cascade (PTGS2 core); The insulin-like growth factor pathway (GO-BP) targets IGF1R to improve cartilage metabolism disorders and inhibit BCL2L1/PIK3R1 mediated cell death during apoptosis response; EGFR resistance and AGE-RAGE pathway elucidate the mechanisms of OA chronicity and glycosylation damage, while fluid shear pathway links mechanical stress-PI3K activity cartilage degeneration; prostaglandin/leukotriene aging metabolism (Wikipathway) is a novel intervention target. The multidimensional network (“hypoxia inflammation metabolism apoptosis” axis) systematically analyzes the integrated biological basis of BYHWD in reversing OA pathology.

### 3.5. Immunomodulation of BYHWD for the treatment of OA

**Figure 9.**
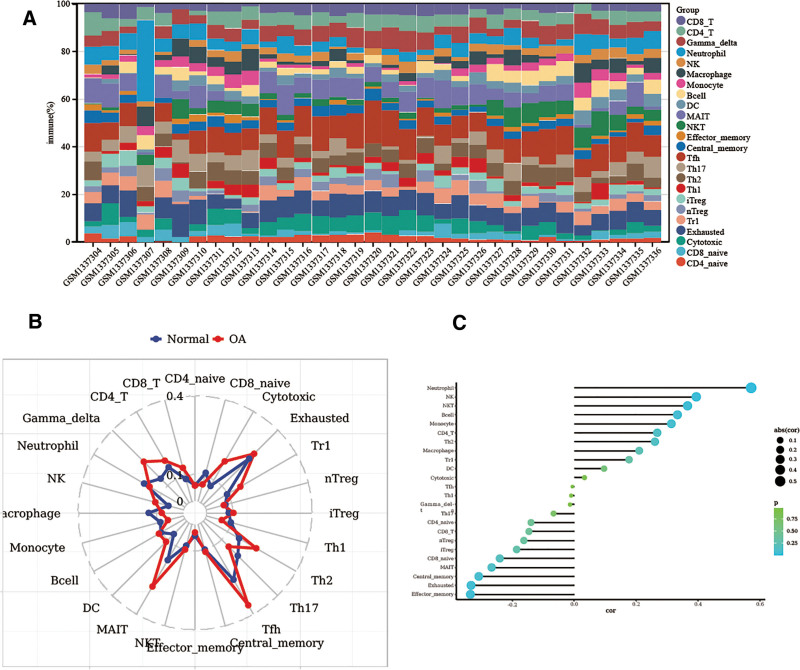
(A) Immunostacking plot of transcriptomic data. (B) Immunoreadar plot of transcriptomic data. (C) Correlation dot-bar plot of transcriptomic data.

Using the ImmuneCellAI algorithm, we were able to determine the immune infiltration score for OA. The findings of this score were displayed in a stacked graph (Table S10, Supplemental Digital Content, https://links.lww.com/MD/Q557, Fig. 9A). The immune infiltration stacked graph revealed that Th1, Th2, Th17, Tfh, DC, B cells, monocytes, macrophages, NK, and CD4_T were the primary immune cells implicated in OA.

The distribution of immune cell data was compared and demonstrated using radar plots. The graphical trends of neutrophil, Th1, Th2, monocyte, macrophage, NK, and other immune cell types showed significant differences between the OA and normal groups, indicating that these immune cells are significantly infiltrated during the development of OA and contribute to the disease (Fig. 9B). Ultimately, correlation dot-bar graphs demonstrated that the infiltration of immune cells displayed varying correlations: CD4_naïve, Gamma_delta, Effector_memory, iTreg, Central_memory, etc demonstrated negative correlations, whereas neutrophil, NK, NKT, monocyte, macrophage, B cell, etc showed positive correlations (Fig. 9C). In conclusion, OA is associated with altered immune cell infiltration patterns, and BYHWD may modify the associated immune cells to treat OA. This study revealed the existence of immune cell infiltration imbalance in OA through the Immune Cell AI algorithm: pro-inflammatory immune subgroups (neutrophils, monocytes/macrophages, NK cells) were significantly increased in the OA group, which directly drove cartilage inflammation and degradation by releasing factors such as IL-1β/TNF-α; adaptive immune disorder: Th1/Th2/Th17/Tfh cell ratio imbalance, mediating autoimmune response and amplifying synovial injury; Immune regulation deficiency: Treg cells (iTreg) and CD4+ memory cells (Central’ memory) are negatively correlated (*P < .05), indicating immune suppression dysfunction. BYHWD reverses the vicious cycle of “pro-inflammatory infiltration immune dysregulation” by regulating the reprogramming of immune cells mentioned above, providing immunological evidence for the treatment of OA.

## 4. Discussion

OA is a prevalent, long-term, debilitating illness that impacts hundreds of millions of individuals globally.^[28]^ A unique type of connective tissue with a limited potential for regeneration due to its inherent lack of vascularity, articular cartilage is the most prominent feature of OA. OA may be prevented and treated with new concepts thanks to recent research demonstrating the importance of HIF-1α and HIF-2α in HIFs as critical regulatory genes for preserving cartilage homeostasis.^[29,30]^ Thus, a novel focus for the therapy of OA involves restoring vascular damage in articular cartilage caused by the HIF signaling system.^[30]^ Wang Qingren, a well-known physician of the Qing Dynasty, described the BYHWD in his book “Medical Forests, Corrections and Errors – Volume II – Paralytic Fistulae.” Blood stasis is essentially caused by abnormal blood rheology, which can be greatly relieved by tonifying Yang Hui Wu Tang. This also has the effect of reducing blood viscosity, preventing red blood cell aggregation, and increasing blood circulation. The latter 2 are its antihypertensive and anti-inflammatory properties. Pharmacological research indicates that tonifying Yang Hui Wu Tang can effectively help characterize the treatment of OA by reducing inflammation, inhibiting capillary permeability, reducing congestion and edema, alleviating the infiltration of inflammatory cells, reducing the synovial layer, and inhibiting the proliferation of synovial cells.^[31]^ We suggest using transcriptome and TCM data to investigate the molecular mechanism of BYHWD for OA in further detail, building upon these foundations.

Using the herbal database and incoming blood component data, we screened for the active ingredients of BYHWD in this study. The 8 key chemicals found in the data are astragaloside, kaempferol, campesterol, stearic acid, succinic acid, nicotinic acid, and formononetin. We identified the major genes associated with OA occurrence by examining the GEO data; these genes included up-regulated SLC29A3, HAUS4, GIMAP4, and down-regulated SLC7A, NUMA1, and ZBTB7C. The BYHWD treatment of the OA process is associated with the immune system, endocrine system, cell growth and death, and signaling, according to the results of KEGG. Its particular molecular bioinformatic process involves pathways like the Rap1 signaling pathway, the HIF-1 signaling pathway, the VEGF signaling pathway, and the EGFR tyrosine kinase inhibitor resistance pathway. Lastly, immunoassays demonstrated that the OA and normal groups differed in terms of neutrophils, Th2, monocyte, macrophage, and NK. Dot-bar plots showed that CD4_naïve, Gamma_delta, Effector_memory, iTreg, and Central_memory showed negative correlations, while neutrophils, NK, NKT, monocyte, macrophage, and B cell showed positive correlations. Finally, BYHWD might influence associated immune cell treatment OA.

The primary constituents of BYHWD comprise glycosides, proteins, amino acids, and other constituents that have the ability to counteract thrombus formation, enhance hemodynamics, impede apoptosis, and mitigate the inflammatory reaction by limiting the release of inflammatory mediators.^[31]^ It has been discovered that BYHWD controls the production of the proteins Bax and Bcl-2, which inhibits chondrocyte apoptosis. Some investigations have established that BYHWD can inhibit thrombus formation by controlling TNF, IL-17, and other signaling pathways, with the NF-κB pathway serving as the central mechanism, based on the findings of network pharmacology. According to some research, BYHWD also improves blood rheology, inhibits platelet aggregation, protects neurons, encourages the creation of myelin sheaths, and aids in the healing of damaged nerves. All things considered, BYHWD has anti-infective, analgesic, circulatory enhancing, and function-restoring qualities.^[32]^ Astragalus is an effective anti-infective, pain reliever, blood stasis eliminator, and nerve function protector. Modern pharmacological studies have demonstrated that the total flavonoids in astragalus can inhibit the overphosphorylation of p38 and JNK proteins in the MAPKs signaling pathway, reduce the content of factors such as iNOS and COX-2, and thus inhibit the occurrence of inflammatory reactions. Additionally, astragaloside protects neuronal regenerative cells by activating the HIF-1α pathway.^[33,34]^ The polysaccharides found in Angelica sinensis have the ability to limit the inflammatory response, lessen the release of inflammatory mediators like TNF-α, IL-6, and IL-1β, and block associated proteins in the NF-κB, MAPK, and STAT signaling pathways.^[35]^ Furthermore, polysaccharides derived from Angelica sinensis have the ability to shield articular chondrocytes by suppressing the development of matrix metalloproteinases MMP-1, MMP-9, and MMP-13.^[36]^ The idea that Dillon is antithrombotic is supported by the fact that its enzymes have been shown to have positive effects on circulation and fibrinolysis. Dilong can also aid in the damaged nerve cells’ ability to heal.^[37]^ The effects of Paeonia lactiflora in stimulating blood circulation have been demonstrated by the discovery that Paeonenolides A and B can inhibit coagulation factor activity, raise nitric oxide levels, promote vasodilation, and counteract thrombosis. Additionally, Paeonenolides A in Paeonia lactiflora has been shown to protect neural cells, further supporting its anti-thrombosis and circulation-improving properties. Collectively, they demonstrated the antithrombotic, circulation-improving, and nerve-cell-protecting properties of Paeonia lactiflora. Huangqi total flavonoids/Danggui polysaccharides synergistically inhibit the MAPKs (p38/JNK)-NF-κB signaling axis, downregulate inflammatory factors such as TNF-α, IL-6, IL-1β, and COX-2/iNOS expression, block synovitis storm, and Paeonolides of Paeonia lactiflora increases NO mediated vasodilation, synergistically antagonizes thrombus formation with earthworm fibrinolytic enzyme, and improves joint microcirculation disorders. Huangqi glycoside activates the HIF-1α-VEGF pathway, promotes hypoxia adaptive angiogenesis, and co regulates the STAT3 pathway with Angelica polysaccharides, reducing MMP-1/9/13 secretion and inhibiting cartilage matrix degradation; BYHWD core components (such as kaempferol and paeoniflorin) target the IGF1R-PI3K/AKT pathway, reverse insulin resistance metabolic disorders, and regulate Bcl-2/Bax balance to inhibit chondrocyte apoptosis. Astragaloside IV/Paeonolides promote nerve regeneration through the HIF-1α-NGF axis, while Angelica polysaccharides regulate Treg/Th17 balance to alleviate autoimmune injury; earthworm enzymes degrade fibrin deposits, relieve nerve compression, and restore motor function. In summary, BYHWD utilizes NF-κB/MAPKs as the inflammatory regulatory hub, HIF-1α-VEGF as the vascular/neural repair axis, and IGF1R-Bcl-2 as the metabolic/apoptotic balance pivot, forming a cascade therapeutic network of “synovitis regression microcirculation reconstruction → cartilage metabolism homeostasis recovery.” This model elucidates that TCM formulas break the vicious cycle of “hypoxia inflammation degeneration” in OA through multi-target spatiotemporal synergy, providing an integrated pharmacological paradigm for the treatment of complex arthritis.

Finally, we discovered that the active components of BYHWD, whose underlying molecular mechanism is closely linked to the vascular process involved in the HIF-1α pathway, might treat OA by modifying the inflammatory response of chondrocytes and thrombus status, thereby improving synovitis. A study has found that immune cells drive OA through the hypoxia inflammation axis, macrophages/Th17 infiltrate and release IL-1β/TNF-α, activate the stabilization of HIF-1α in synovial cells, induce abnormal high expression of VEGF, pathological angiogenesis, exacerbate subchondral bone remodeling abnormalities and synovitis edema. HIF-1α simultaneously promotes Th17 differentiation and inhibits Treg function through positive feedback, amplifying immune imbalance. In addition, VEGF and PTGS2 synergistically upregulate MMP-9/13, directly degrading the cartilage matrix.^[9,38–40]^ BYHWD may inhibit the “immune hypoxia vascular disruption” cycle and simultaneously improve the immune microenvironment and metabolic homeostasis through Astragaloside IV/Angelica polysaccharides.

Our current study has many limitations. Firstly, more research is required to fully understand the intricate structure of the herbal substance and the molecular mechanisms underlying each of the constituent chemical parts of OA. Secondly, in light of the current research, we did not perform ex vivo investigations, which we will do in later work. This study has the following computational biology limitations: database dependency bias: TCMSP/ITCM does not cover all components of TCM (such as earthworm enzymes), and ITCM data has not been standardized experimentally, which may omit key active substances; algorithm resolution limitation: immune cell AI is based on transcriptome deconvolution, which results in insufficient recognition accuracy for rare immune subgroups such as gamma delta T cells; network fraud positive: PPI and co-expression analysis are difficult to distinguish direct/indirect effects, and target association needs to be validated through wet experiments; oversimplification of pathway enrichment: KEGG/Wikipathway does not reflect the OA tissue-specific pathway Crosstalk, which may weaken the complexity of the mechanism. Lastly, we think that a key area for future study will be the regulation of inflammatory alterations in the synovium in OA based on the HIF-1α pathway.

## 5. Conclusion

Using transcriptome data in conjunction with online TCM information, we conducted an analysis and summary of the immunological and inflammation-related molecular pathways of OA in this study. Additionally, we identified possible therapeutic components and targets of action of BYHWD for the treatment of OA. The primary active components of BYHWD, astragaluside, kaempferol, paeoniflorin, and formononetin, act via the key genes controlling the Rap1 signaling pathway, EGFR tyrosine kinase inhibitor resistance, and HIF-1/VEGF signaling pathway to ameliorate OA. The development and management of OA synovitis may be significantly impacted by the aforementioned signaling pathways, which merit further investigation in the future research. Despite the limitations of machine analysis methods, in-depth study of the complex mechanisms that produce therapeutic outcomes will provide reasons for the application of TCM.

## Author contributions

**Conceptualization:** Qi Liang.

**Data curation:** Xue Li.

**Investigation:** Ying Lv.

**Methodology:** Siyu Wang.

**Software:** Xingyong Yang.

## Supplementary Material


